# Density Matrix Embedding Using Multiconfiguration Pair-Density Functional Theory

**DOI:** 10.1021/acs.jctc.3c00247

**Published:** 2023-06-06

**Authors:** Abhishek Mitra, Matthew R. Hermes, Laura Gagliardi

**Affiliations:** † Department of Chemistry, Chicago Center for Theoretical Chemistry, University of Chicago, Chicago, Illinois 60637, United States; ‡ Department of Chemistry, Pritzker School of Molecular Engineering, James Franck Institute, Chicago Center for Theoretical Chemistry, University of Chicago, Chicago, Illinois 60637, United States; ¶ Argonne National Laboratory, 9700 S. Cass Avenue, Lemont, Illinois 60439, United States

## Abstract

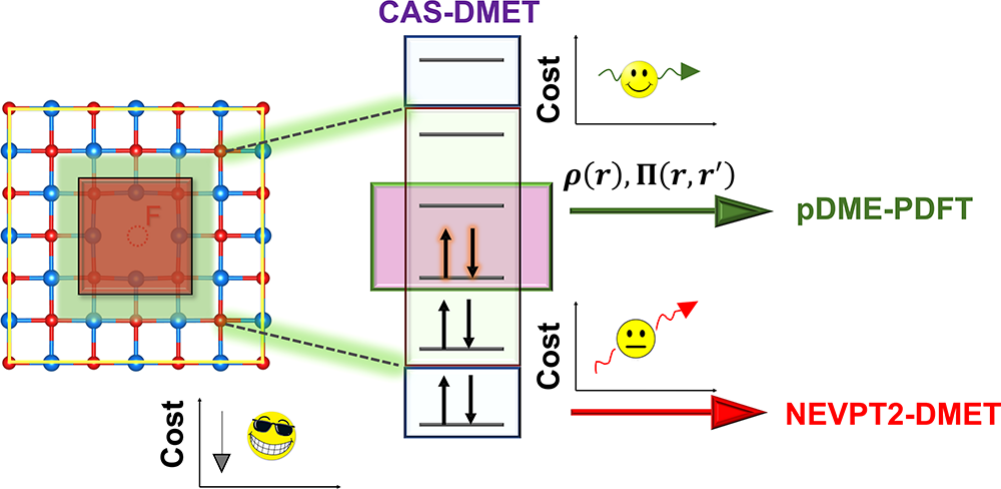

We present a quantum embedding method for ground and excited states of extended systems that uses multiconfiguration pair-density functional theory (MC-PDFT) with densities provided by periodic density matrix embedding theory (pDMET). We compute local excitations in oxygen mono- and divacancies on a magnesium oxide (100) surface and find absolute deviations within 0.05 eV between pDMET using the MC-PDFT, denoted as pDME-PDFT, and the more expensive, nonembedded MC-PDFT approach. We further use pDME-PDFT to calculate local excitations in larger supercells for the monovacancy defect, for which the use of nonembedded MC-PDFT is prohibitively costly.

## 1 Introduction

Quantum embedding methods are promising for accurately describing electron correlation in molecules and materials, especially when correlated wave function methods become prohibitively expensive due to their poor scaling with system size.^1−11^ These methods involve dividing a system into important regions (called impurities or fragments) that are treated with a highly correlated theory, while the rest of the system is described using a more approximate level of theory, such as Hartree–Fock (HF)^12^ or Kohn–Sham density functional theory.^13,14^ One particular type of quantum embedding method is density matrix embedding theory (DMET),^2,15−17^ which uses a wave function-in-wave function approach and models the environment of the impurity or fragment using a bath constructed from the Schmidt decomposition^18^ of a mean-field wave function.

For systems with significant static (strong) correlation, multiconfiguration methods are often used to describe the ground and excited states of molecular systems. The complete active space self-consistent field (CASSCF) method^19−21^ expresses the wave function as a linear combination of all possible configuration state functions that can be generated within a defined “active space” of *n* active electrons occupying *N* active orbitals. To get accurate electronic excitation energies and reaction energies, post-SCF methods such as the complete active space second-order perturbation theory (CASPT2)^22^ or n-electron valence state second-order perturbation theory (NEVPT2)^23−26^ can be used, as well as multiconfiguration pair-density functional theory (MC-PDFT).^27−30^

Multiconfiguration methods are desired as high-level (impurity) solvers in DMET because they can handle extended systems with multiple electronic configurations.^31−33^ Recently n-electron valence state second-order perturbation theory (NEVPT2) was implemented as a high-level quantum chemical solver within periodic DMET (pDMET) to capture dynamic correlation as a post-CAS-DMET procedure.^32^ However, even though NEVPT2-DMET is cheaper than NEVPT2, it scales poorly with the active space size and the parameter space (i.e., the number of orbitals in the impurity).^32^ A more affordable alternative for capturing electron correlation at the post-SCF level is multiconfiguration pair-density functional theory (MC-PDFT)^27,29,30^ and its hybrid version (HMC-PDFT).^34^ In a recent benchmark study of 373 vertical excitation energies from the QUESTDB data set, HMC-PDFT was found to be as accurate or even more accurate than NEVPT2 for excitation energies.^35^

Here, we present a way to calculate the correlation energy starting from a CAS-DMET wave function using PDFT and hybrid PDFT. Our implementation is designed for systems with periodic boundary conditions (extended systems), specifically inspired by the class of problems we are tackling, such as point defects in crystals. It can be easily adapted to molecular systems with open boundary conditions. Here onward, we refer to this approach as pDME-PDFT and we employ it to calculate singlet–singlet and singlet–triplet excitation energies in the F and M centers on the (100) surface of magnesium oxide. F-centers play an important role in catalysis,^36^ energy storage,^37^ and photoelectrochemical applications^38−40^ and are responsible for several physical and chemical properties of MgO.^41^ M-centers are an aggregate of two adjacent F-centers, which also affect the physical and chemical properties of MgO, such as its electrical conductivity, magnetic behavior, and optical properties.^41^

## 2 Theory

### 2.1 Multiconfiguration Pair-Density Functional Theory (MC-PDFT)

The MC-PDFT energy for a multiconfiguration (MC) wave function is expressed as
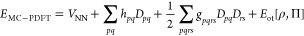
1Here, *V*_NN_ is the nuclear–nuclear repulsion energy, *p*, *q*, *r*, and *s* denote molecular orbitals, *h*_*pq*_ and *g*_*pqrs*_ are one- and two-electron integrals, *D*_*pq*_ are the elements of the one-electron reduced density matrices (1-RDMs), and *E*_ot_ is a functional of the density (ρ) and the on-top pair-density (Π). The hybrid MC-PDFT energy^34^ is expressed as
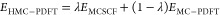
2Here, *E*_MCSCF_ is the energy derived from the MC wave function in use and λ is the hybrid parameter which specifies the percentage of MCSCF energy included in the hybridization. Our calculations were performed using a λ value of 0.25, referred to as tPBE0, in analogy with the PBE0 hybrid density functional theory (DFT) functional.^34,42^

### 2.2 Periodic Density Matrix Embedding Theory (pDMET) and the pDME-PDFT Implementation

DMET and its periodic implementation have been discussed in detail previously.^2,15−17,32,43−48^ DMET involves a low-level (usually Hartree–Fock) calculation on a whole system followed by a high-level (in our case, CASSCF or NEVPT2) calculation in an unentangled “embedding” subspace consisting of the union of user-specified fragment orbitals and corresponding bath (i.e., entangled environment) orbitals identified using the Schmidt decomposition.^16^ The 1-RDM and two-body reduced density matrix (2-RDM) of the whole system consist of the 1- and 2-RDMs, respectively, from the high-level calculation in the embedding subspace combined with those from the low-level calculation in the orthogonal “core” subspace. If (as in this work) only one embedded fragment is considered in each calculation and the low-level wave function (here, restricted open-shell HF, ROHF) is spin-symmetry-adapted and closed-shell in the core subspace, then the expressions for the DMET whole-system 1- and 2-RDMs assume the simple forms:
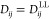
3a
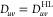
3b
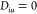
3c
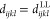
3d
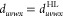
3e
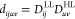
3f
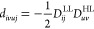
3g
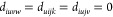
3hwhere indices *i*, *j*, *k*, *l* and *u*, *v*, *w*, *x* indicate core and embedding orbitals, respectively, and superscripts LL and HL indicate low-level and high-level calculations, respectively. (N.B.: the 1- and 2-RDMs have the index-permutation symmetries *D*_*pq*_ = *D*_*qp*_ and *d*_*pqrs*_ = *d*_*qpsr*_ = *d*_*rspq*_, respectively.) Less generally but more simply, the superscripts LL and HL in eqs 3 can be ignored, and the indices *i*, *j* and *u*, *v* can instead be taken to identify doubly occupied inactive orbitals (in either the embedding or core subspace) and active orbitals (which must be in the embedding subspace), respectively, since the 1- and 2-RDM elements for doubly occupied orbitals are trivial (*D*_*ij*_ = 2δ_*ij*_ and *d*_*ijkl*_ = 4δ_*ij*_δ_*kl*_ – 2δ_*il*_δ_*jk*_).

The density and the on-top pair-density are calculated from the 1-RDMs and 2-RDMs obtained from eqs 3 using the formulas:
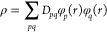
4
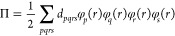
5and substituted into eq 1. When using hybrid MC-PDFT, the first term of eq 2 (*E*_MCSCF_) is taken as the CAS-DMET total energy. In comparing nonembedded MC-PDFT and pDME-PDFT calculations, the key difference lies in the origin of the 1- and 2-RDMs: for MC-PDFT, they are obtained from a nonembedded CASSCF calculation, while for pDME-PDFT, they are derived from a CAS-DMET calculation, the computational cost of which is directly proportional to the size of the embedding space. The computational savings achieved with CAS-DMET over the nonembedded CASSCF stem from freezing core orbitals and optimizing only the fragment and bath orbitals, in contrast to a conventional full-system CASSCF calculation, which optimizes the entire orbital space (here, HF orbitals). This results in fewer electronic degrees of freedom for CAS-DMET compared to CASSCF.^31,32^

Note that the implementation of pDME-PDFT differs from the one of NEVPT2-DMET in the following way: while pDME-PDFT evaluates the total energy using the density and on-top pair-density of the whole system (see eqs 4 and 5), NEVPT2-DMET applies the NEVPT2 method only to the embedding space. Since pDME-PDFT is agnostic to the way in which the embedding calculation has been performed, it is designed to recover in part the effects of dynamic electron correlation even for inactive electrons, which are not correlated in the underlying trial wave function. In contrast, NEVPT2-DMET can not describe electron correlation beyond the embedding space. Moreover, pDME-PDFT has a lower cost scaling with respect to the embedding space size compared to NEVPT2-DMET, making it potentially more advantageous both in terms of accuracy and cost reduction. It is worth noting that the 1- and 2-RDMs are influenced by the core/inactive environment, which means that the results of pDME-PDFT calculations may be affected by the choice of the mean-field method used to define the core or inactive space in each embedding calculation.

## 3 Computational Methods

All the DMET calculations were performed using our in-house pDMET and mrh codes^49,50^ which utilizes the electron integrals and quantum chemical solvers from PySCF.^51,52^ Wannierization was done using the wannier90^53^ code via the pyWannier90 interface.^54^ The Wannierization step involves constructing maximally localized Wannier functions (MLWFs)^55,56^ from the ROHF molecular orbitals. These localized orbitals are used to select the impurity subspace, followed by a Schmidt decomposition of the impurity-environment block of the 1-RDM to generate entangled bath orbitals. The impurity and entangled bath space form the embedding space where high-level electronic structure solvers like CASSCF are used. The details about the CAS-DMET steps can be found in ref (32). The Goedecker–Teter–Hutter pseudopotentials^57,58^ were used for all the calculations. The geometry optimizations were performed at the spin-unrestricted PBEsol level^59^ using the Vienna *Ab initio* Simulation Package (VASP).^60−63^ The convergence criteria of 10^–6^ eV and 10^–3^ eV/Å were used for the energy and force, respectively. We represent a MgO(100) surface using a single layer of Mg and O with the chemical formula Mg_18_O_18_. We performed benchmark calculations on two point defects, namely, the oxygen monovacancy (OV) and a oxygen divacancy (OOV). For these systems, we computed singlet–singlet and singlet–triplet excitation energies using CAS-DMET, NEVPT2-DMET, and pDME-PDFT. We used the translated PBE functional for both PDFT and hybrid PDFT which are referred to as pDME-tPBE and pDME-tPBE0, respectively. The oxygen monovacancy defect is created by removing one neutral oxygen atom at the center of the unit cell. The divacancy is created by removing an additional neutral oxygen atom nearest to the monovacant oxygen atom. To separate the layer and its periodic images, we used a vacuum of 23.518 Å along the [100] direction. In the DMET calculations, we place a dummy oxygen atom at the vacancy to provide basis functions to span the electron density of the defect. For the monovacancy, the dummy oxygen and four nearest Mg atoms are treated using the polarized triple-ζ basis set (GTH-TZVP) whereas the rest of the atoms are treated with the polarized double-ζ basis set (GTH-DZVP). For the divacancy, the dummy oxygens and six nearest Mg atoms are treated using the polarized triple-ζ basis set (GTH-TZVP) whereas the rest of the atoms are treated with the polarized double-ζ basis set (GTH-DZVP). The two and three layered models are constructed by placing the nondefective one and two layers of Mg_18_O_18_ below the first layer, respectively. For these models the GTH-TZVP is used for the dummy oxygen and nine nearest atoms (4 O and 5 Mg) while GTH-DZVP is used for all other atoms.

## 4 Results and Discussion

**Figure 1:**
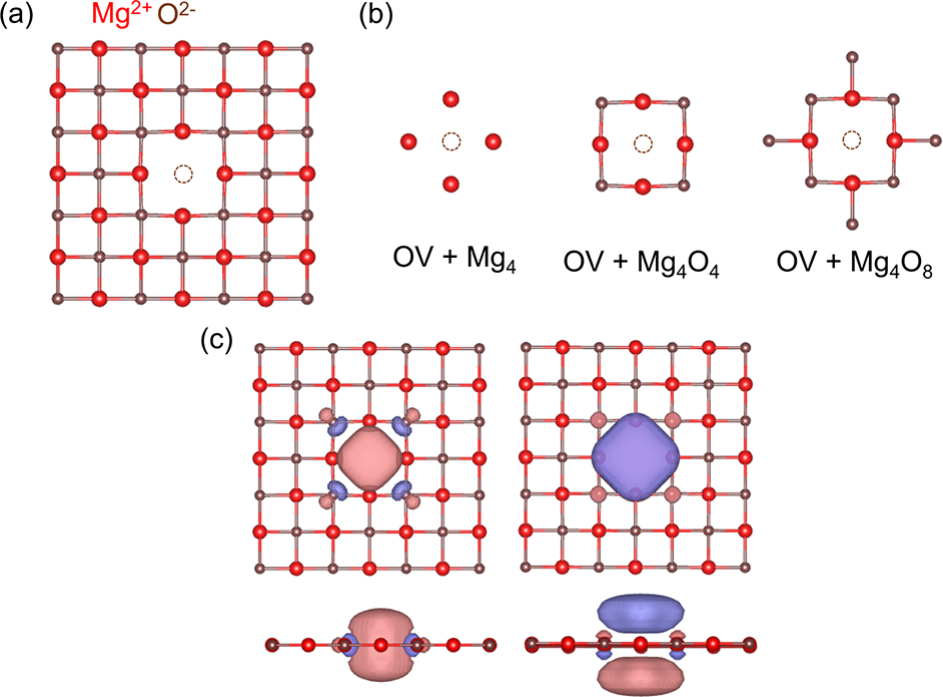
Oxygen monovacancy on a Mg_18_O_18_ layer: (a) Top view of the F-center on the (100) surface. (b) Three different impurity clusters considered in the DMET calculations. (c) Top and side views of two defect natural orbitals from the converged CASSCF calculation considered in the (2, 2) active space. The isosurface of the orbitals is 0.03. This figure has been adapted from ref (32).

**Figure 2:**
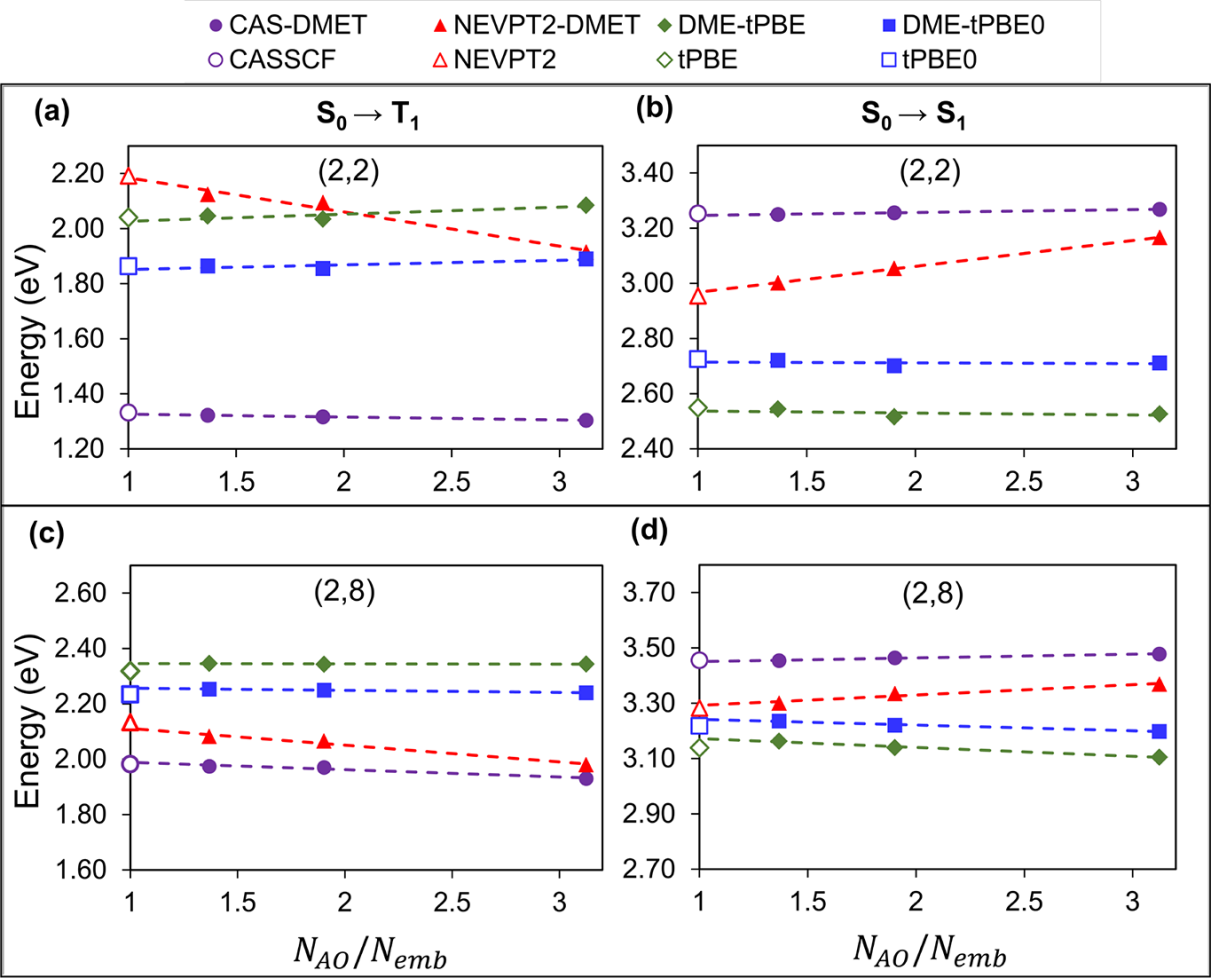
Excitation energies of the OV defect in the Mg_18_O_18_ layer using an ROHF bath and active spaces of (2,2) and (2,8) calculated by CAS-DMET (purple circles), NEVPT2-DMET (red triangles), pDME-tPBE (dark green diamonds), and pDME-tPBE0 (blue squares) for S_0_ → T_1_ (a, c) and S_0_ → S_1_ (b, d) excitations as a function of *N*_AO_/*N*_emb_. Dashed lines are used for extrapolation, and reference energies from CASSCF (purple), NEVPT2 (red), tPBE (dark green), and tPBE0 (blue) are shown for comparison. *N*_AO_ is the total number of basis functions in Mg_18_O_18_, and *N*_emb_ is the number of embedding orbitals in the impurity clusters considered. Here *N*_AO_ is 506.

First, we investigate the performance of pDME-PDFT in calculating the S_0_ → S_1_ and S_0_ → T_1_ excitations of the F-center which is a neutral oxygen monovacancy (OV) on the (100) monolayer of MgO. Experimentally, detecting F-centers on MgO surfaces presents a challenge due to its surface sensitivity, resulting in a range of S_0_ → S_1_ transitions observed between 1 and 5 eV as reported in Table 1.^64−67^ A quantum mechanics/molecular mechanics (QM/MM) approach, utilizing the multireference configuration interaction method, for a cluster model of the oxygen monovacancy predicted excitation energies of 3.24 eV for the S_0_ → S_1_ transition and 1.93 eV for the S_0_ → T_1_ transition.^68^ The MgO lattice is composed of Mg^2+^ and O^2–^ ions, and when an oxygen atom is removed, it leaves behind two electrons in the defect site that occupy two defect-localized states between the valence band maximum (VBM) and the conduction band minimum (CBM). The computational model is illustrated in Figure 1a. To examine how the excitation energies vary with the embedding space, we consider three impurity clusters of increasing size, as depicted in Figure 1b. Figure 1c shows the two active natural orbitals used for the minimal (2,2) active space in all calculations presented in Figure 2a,b. This active space has been used previously for the F-center.^32,64^ The two active orbitals have a_1g_ and a_2u_ symmetry in the D_4h_ point group. The natural orbitals shown in Figure 1c are obtained from the converged nonembedded CASSCF calculations (used in the subsequent MC-PDFT calculations). The natural orbitals derived from the embedded CAS-DMET calculations, which are employed in the corresponding pDMET-PDFT calculations, qualitatively represent the same active space.

**Table 1:** Vertical Excitation Energies (in eV) of the Oxygen Monovacancy on the Mg_18_O_18_ Layer obtained Using DMET with CASSCF, NEVPT2, MC-PDFT (tPBE), and HMC-PDFT (tPBE0)^a^

Excitation	Active space	Impurity cluster	CASSCF	NEVPT2	tPBE	tPBE0	Literature
*S*_0_ → *T*_1_	(2,2)	OV+Mg_4_	1.30	1.91	2.09	1.89	
		OV+Mg_4_O_4_	1.32	2.09	2.03	1.86	
		OV+Mg_4_O_8_	1.32	2.12	2.05	1.87	
		Extrap	1.33	2.18	2.03	1.85	
	Reference		1.33	2.19	2.04	1.86	
	(2,8)	OV+Mg_4_	1.93	1.98	2.34	2.24	1.93 (MRCI)^68^
		OV+Mg_4_O_4_	1.97	2.07	2.34	2.25	
		OV+Mg_4_O_8_	1.97	2.08	2.35	2.25	
		Extrap	1.99	2.18	2.35	2.26	
	Reference		1.98	2.13	2.32	2.23	
*S*_0_ → *S*_1_	(2,2)	OV+Mg_4_	3.27	3.17	2.53	2.71	
		OV+Mg_4_O_4_	3.26	3.05	2.52	2.70	
		OV+Mg_4_O_8_	3.25	3.00	2.54	2.72	
		Extrap	3.25	2.97	2.54	2.71	
	Reference		3.25	2.95	2.55	2.72	3.24 (MRCI)^68^
	(2,8)	OV+Mg_4_	3.48	3.37	3.11	3.20	2.30 (Exp)^65^
		OV+Mg_4_O_4_	3.46	3.34	3.14	3.22	1.0, 1.3, 2.4, 3.4 (Exp)^66^
		OV+Mg_4_O_8_	3.45	3.30	3.16	3.24	1.2, 3.6, 5.3 (Exp)^67^
		Extrap	3.45	3.29	3.17	3.24	
	Reference		3.45	3.30	3.16	3.24	

a The extrapolated CAS-DMET, NEVPT2-DMET, tPBE-DMET, and tPBE0-DMET energies from the linear regression are labeled as “Extrap”. “Reference” here indicates the non-embedded Γ-point CASSCF, NEVPT2, tPBE, and tPBE0 calculations.

In Figure 2a,b, we show the vertical excitation energies of the S_0_ → T_1_ and S_0_ → S_1_ transitions in the OV system, respectively, as a function of the inverse of the number of embedding orbitals, using the minimal (2,2) active space. Specifically, the plot of excitation energies is shown as a function of *N*_AO_/*N*_emb_ where *N*_AO_ represents the total number of basis functions in the system considered (here Mg_18_O_18_) and *N*_emb_ is the number of embedding orbitals in the impurity clusters considered. We compare them to the corresponding nonembedded results represented by hollow markers. The values are reported in Table 1. The excitation energies computed using pDME-tPBE and pDME-tPBE0 agree to within 0.06 eV of the nonembedded reference values for all impurity clusters considered. NEVPT2-DMET, on the other hand, shows a higher sensitivity to the impurity cluster. This is expected since NEVPT2-DMET cannot describe electron correlation outside the embedding space. Considering the S_0_ → T_1_ gap, for example, the NEVPT2-DMET difference with respect to the nonembedding reference ranges from 0.17 to 0.05 eV. As previously done for NEVPT2-DMET,^32^ the linear dependence of the excitation energies with respect to the inverse of the number of embedding orbitals was utilized to extrapolate the nonembedding limit. Here, the nonembedding limit corresponds to the point where *N*_AO_/*N*_emb_ = 1, *i.e.*, *N*_emb_ = *N*_AO_. All the extrapolated values lie within 0.05 eV of the nonembedding reference. This extrapolation is represented using dashed lines in Figure 2.

In Figure 2c,d, we plot the vertical excitation energies using a (2,8) active space as was used in ref (32). The corresponding numbers are reported in Table 1. The active orbitals are reported in the SI. The excitation energies obtained from various nonembedding correlated theories exhibit closer agreement with one another in the larger (2,8) active space, providing a means of evaluating the performance of DME-PDFT for both smaller (2,2) and larger (2,8) active spaces. For the (2,8) active space, all pDME-tPBE and tPBE0 excitation energies agree to within 0.05 eV of the nonembedding references, whereas NEVPT2-DMET shows a higher (although not very significant) sensitivity to the impurity cluster. To quantify the sensitivity of the excitation energies to the embedding space we report the slopes for all the linear extrapolations in Tables S2 and S3 of the SI.

Reference (32) investigated the impact of the choice of mean-field bath on the accuracy of CAS-DMET and NEVPT2-DMET excitation energies. It was found that the ROHF bath outperformed the RHF bath, and as a result, we have used the ROHF bath for all calculations in this work. Although exploring the sensitivity of pDME-PDFT excitation energies to different low-level mean-field baths is an interesting area of research; it falls outside the scope of this study. It is worth noting that the 1- and 2-RDMs used to construct the densities, as discussed in Section 2.2, are dependent on the inactive/core subspace, which underscores the importance of selecting an appropriate mean-field method.

**Figure 3:**
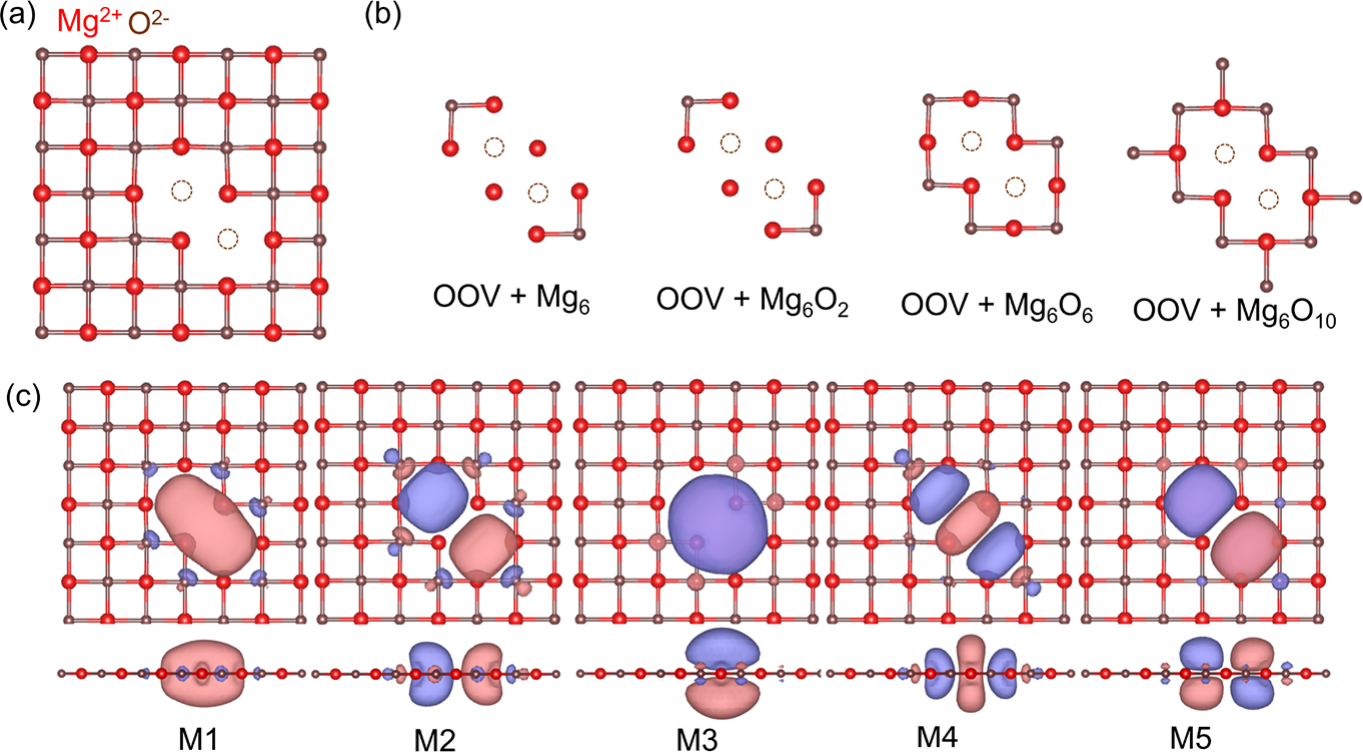
Oxygen divacancy on a Mg_18_O_18_ layer: (a) Top view of the M-center on the (100) surface. (b) Four different impurity clusters considered in the DMET calculations. (c) Top and side views of five defect natural orbitals from the converged CASSCF calculation considered in the (4,5) active space. The isosurface of the orbitals is 0.02.

Next, we investigate the S_0_ → S_1_ and S_0_ → T_1_ excitations of the M-center, which is a neutral oxygen divacancy (OOV) on the (100) monolayer of MgO. This defect is also known as the M-center. Here, the removal of two neutral oxygen atoms leaves four electrons in the cavity created by the two missing oxygens. In the singlet ground state these electrons occupy the two defect-localized states present between the VBM and the CBM.^64^ Experimentally, Kramer et al. tentatively assigned the 1.0 and 1.3 eV adsorption peaks to the M-center on thin films of MgO.^66^ The computational model is shown in Figure 3a. We consider four impurity clusters as shown in Figure 3b. We show the five active natural orbitals forming the minimal (4,5) active space in Figure 3c. The natural orbitals shown in Figure 3c are obtained from the converged nonembedded CASSCF calculations (used in the subsequent MC-PDFT calculations). The natural orbitals derived from the embedded CAS-DMET calculations, which are employed in the corresponding pDMET-PDFT calculations, qualitatively represent the same active space.

**Figure 4:**
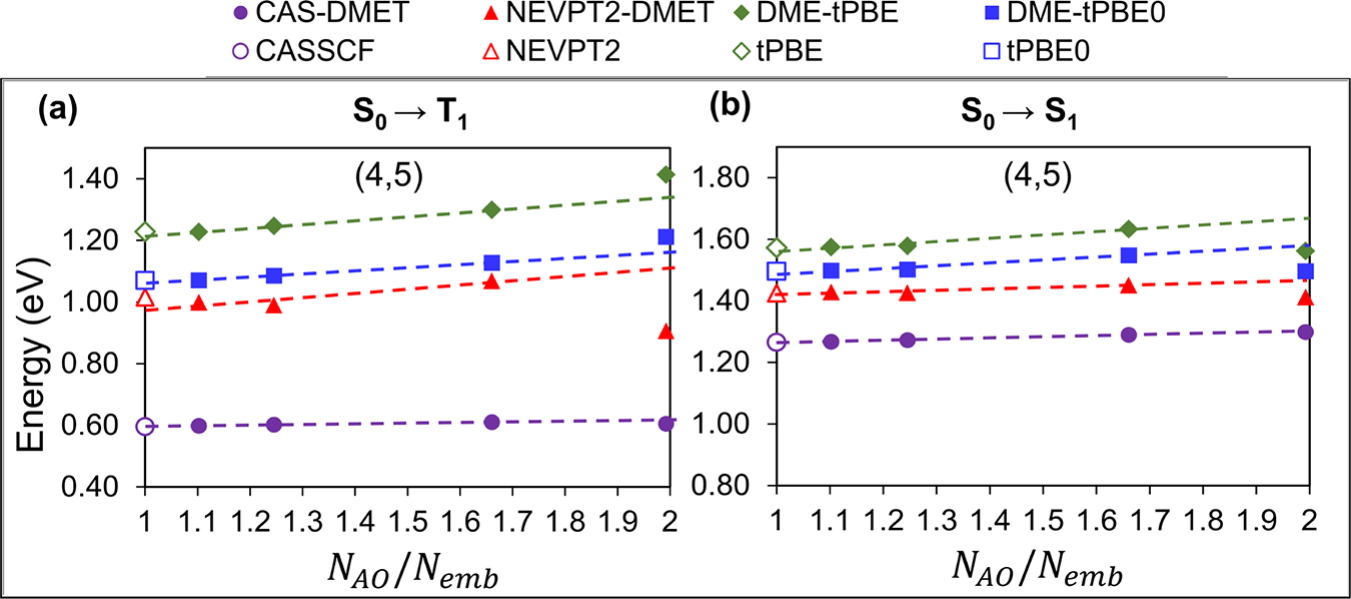
Excitation energies of OV in the MgO layer using the ROHF bath and (4,5) active space for S_0_ → T_1_ (a) and S_0_ → S_1_ (b) calculated by CAS-DMET (purple circles), NEVPT2-DMET (red triangles), pDME-tPBE (dark green diamonds), and pDME-tPBE0 (blue squares) as a function of *N*_AO_/*N*_emb_. Reference energies from CASSCF (purple), NEVPT2 (red), tPBE (dark green), and tPBE0 (blue) are shown for comparison. Here *N*_AO_ is 518.

In Figure 4, we present the vertical excitation energies for the OOV system. The corresponding numbers are reported in Table 2. Although the excitation energies calculated using pDME-tPBE0 for the three larger fragments OOV+Mg_6_O_2_, OOV+Mg_6_O_6_, and OOV+Mg_6_O_10_ are within 0.07 eV of the corresponding nonembedded calculations, the smallest fragment OOV+Mg_6_ deviates by 0.14 eV for the S_0_ → T_1_ gap. This highlights the inadequacy of the smallest impurity cluster (OOV+Mg_6_) in providing an accurate approximation of the overall system densities. Therefore, when extrapolating to the nonembedding limit, only the three larger fragments are taken into account. The excitation energies for the OOV+Mg_6_ impurity cluster clearly fall outside the range of the linear extrapolation, as indicated by the detailed analysis presented in Section S01 of the Supporting Information, which includes *R*^2^ values for the linear fits. The results for the OOV system appear to be slightly more sensitive, as indicated by the slopes of the linear extrapolations in Table S3 of the SI, compared to those of the F-center. The S_0_, S_1_, and T_1_ configurations are primarily composed of the first three active orbitals, represented by M1, M2, and M3 in Figure 3c. These orbitals closely align with the a_1_, b_1_, and a_2_ orbitals in the C_2v_ point group. While the S_0_ state is primarily composed of the M1^2^M2^2^ configuration, both the S_1_ and T_1_ states are dominated by configurations resulting from a M2 → M3 transition.

**Table 2:** Vertical Excitation Energies (in eV) of the Oxygen Divacancy on the MgO(100) Surface Obtained Using CAS-DMET, NEVPT2-DMET, pDME-tPBE, and pDME-tPBE0, with an Active Space of 4 Electrons in 5 Orbitals^a^

Excitation	Layers	Impurity cluster	CASSCF	NEVPT2	tPBE	tPBE0	Literature
*S*_0_ → *T*_1_	Mg_18_O_18_	OOV+Mg_6_	0.61	0.91	1.41	1.21	
		OOV+Mg_6_O_2_	0.61	1.07	1.30	1.13	
		OOV+Mg_6_O_6_	0.60	0.99	1.25	1.09	
		OOV+Mg_6_O_10_	0.60	1.00	1.23	1.07	
		Extrap	0.60	0.97	1.21	1.06	
	Reference		0.60	1.02	1.23	1.07	
*S*_0_ → *S*_1_	Mg_18_O_18_	OOV+Mg_6_	1.30	1.41	1.56	1.50	
		OOV+Mg_6_O_2_	1.29	1.45	1.63	1.55	2.00 (CASPT2)^64^
		OOV+Mg_6_O_6_	1.27	1.43	1.58	1.50	1.19 (TD-DFT)^64^
		OOV+Mg_6_O_10_	1.27	1.43	1.57	1.50	1.0, 1.3 (Exp)^66^
		Extrap	1.26	1.42	1.56	1.48	
	Reference		1.27	1.43	1.58	1.50	

a The extrapolated energies from linear regression of the last three points are labeled as “Extrap”. “Reference” here indicates the non-embedded Γ-point CASSCF, NEVPT2, tPBE, and tPBE0 calculations.

**Figure 5:**
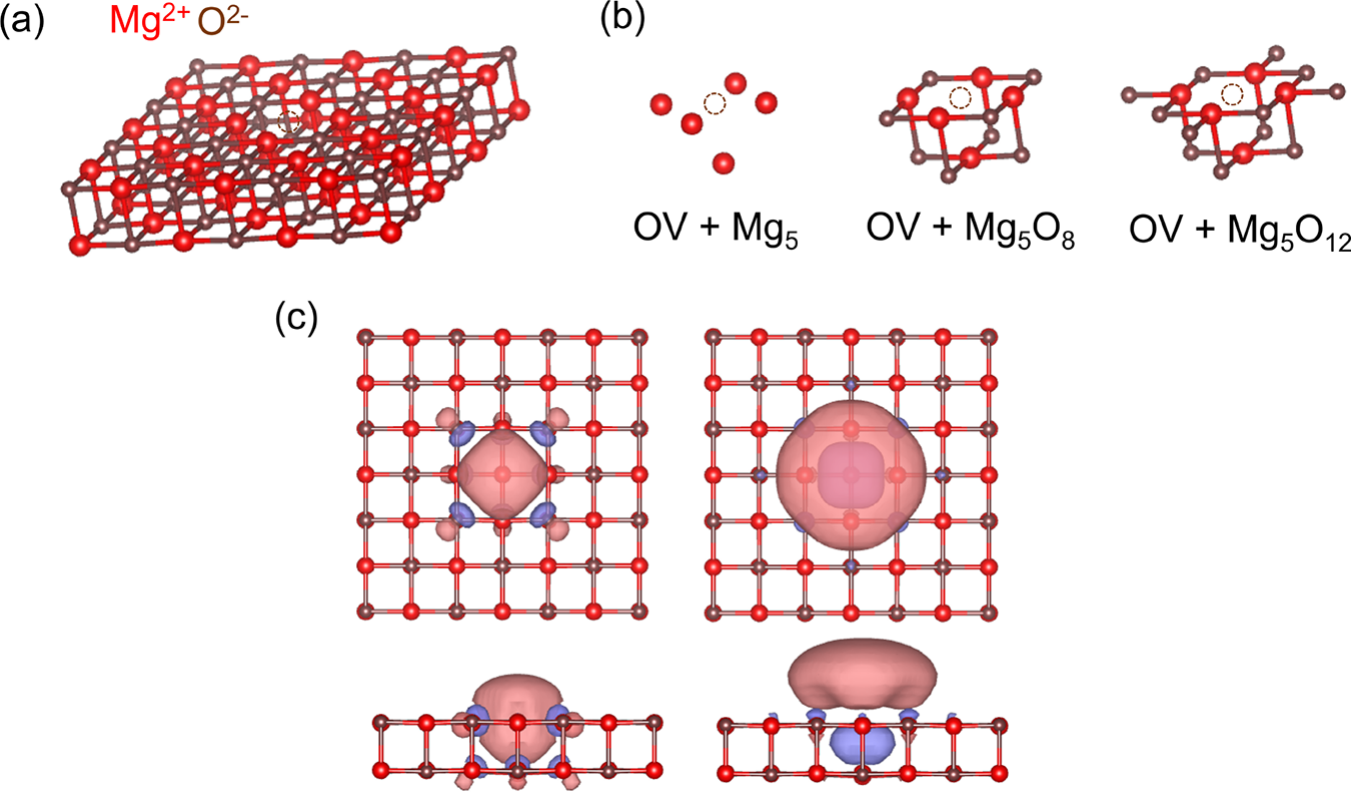
Oxygen monovacancy on a Mg_36_O_36_ surface: (a) F-center on the (100) surface. (b) Three different impurity clusters considered in the DMET calculations. (c) Top and side views of two defect natural orbitals from the converged CAS-DMET calculation considered in the (2,2) active space. The isosurface of orbitals is 0.02.

Next, we explore electronic excitations in the oxygen monovacancy on MgO surfaces containing two and three layers, where the corresponding nonembedding calculations are prohibitively costly. The active spaces used are (2,2) and (2,8). The computational model used for the OV defect in 2 layers of MgO (Mg_36_O_36_), the impurity clusters used in the embedding calculations, and the natural active orbitals in the minimal (2,2) active space are shown in Figure 5a–c, respectively. Since the nonembedding calculations are prohibitive, the natural orbitals shown in Figure 5c are obtained from the largest converged CAS-DMET calculations.

**Figure 6:**
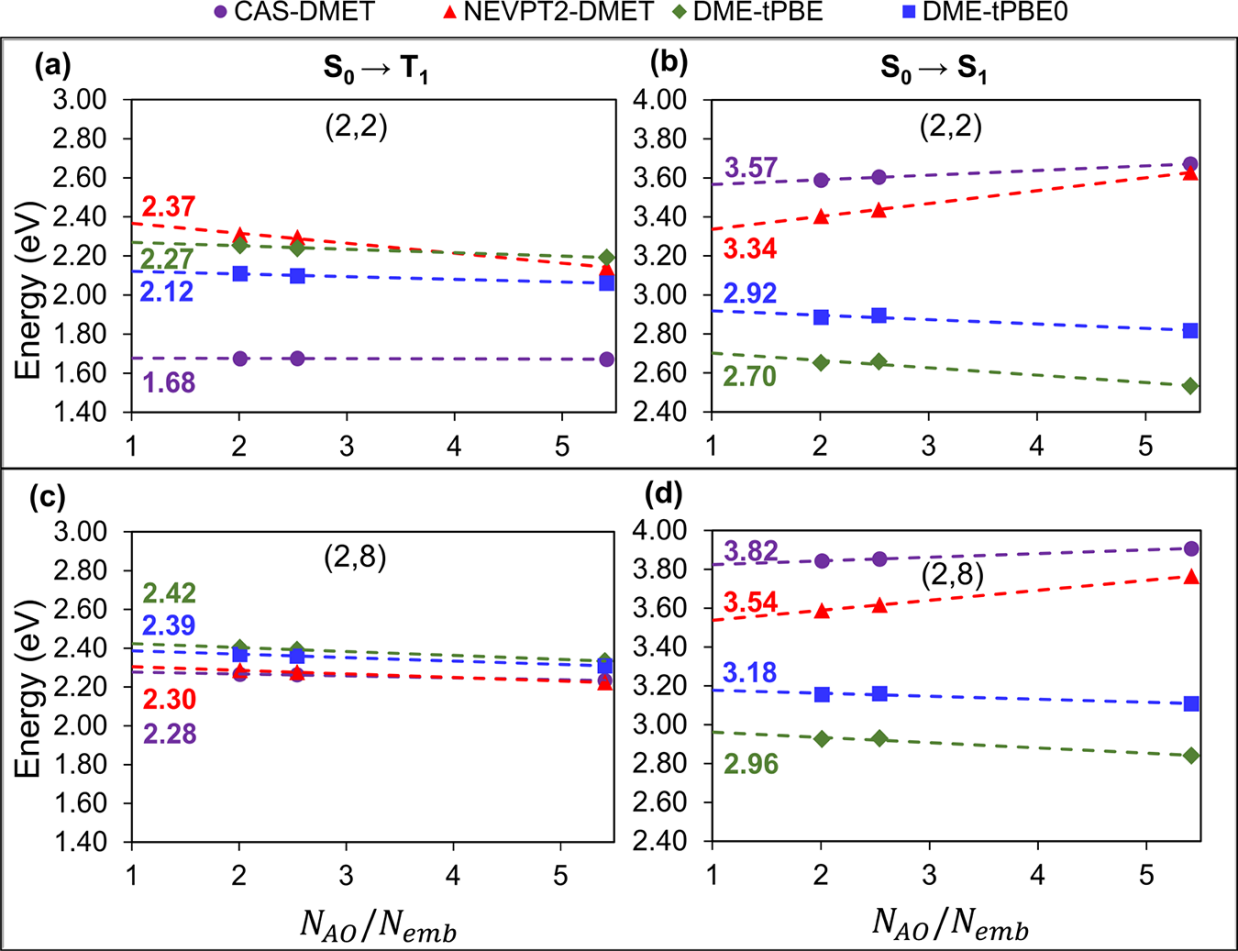
Excitation energies of the OV defect in the Mg_36_O_36_ surface using an ROHF bath and active spaces of (2,2) and (2,8) calculated by CAS-DMET (purple circles), NEVPT2-DMET (red triangles), pDME-tPBE (dark green diamonds), and pDME-tPBE0 (blue squares) for S_0_ → T_1_ (a, c) and S_0_ → S_1_ (b, d) excitations as a function of *N*_AO_/*N*_emb_. All energies are extrapolated to the nonembedding limit; *N*_AO_ represents the number of basis functions and *N*_emb_ is the number of embedding orbitals. Here *N*_AO_ is 996.

The vertical excitation energies for the OV defect in Mg_36_O_36_ obtained from the embedding calculations are plotted in Figure 6 and reported in Table 3. NEVPT2-DMET, pDME-tPBE, and pDME-tPBE0 increase the S_0_ → T_1_ excitation energy and decrease the S_0_ → T_1_ excitation energy as compared to the corresponding CAS-DMET values. The correction is more prominent for the (2,2) active space since CAS-DMET is expected to capture a smaller percentage of the dynamic correlation effects than that of the (2,8) active space. Overall, the extrapolated NEVPT2-DMET and pDME-tPBE0 excitation energies agree within 0.5 eV of each other.

**Table 3:** Vertical Excitation Energies (in eV) of the Oxygen Monovacancy on the Mg_36_O_36_ Surface Obtained Using DMET with CASSCF, NEVPT2, MC-PDFT (tPBE), and HMC-PDFT (tPBE0)^a^

Excitation	Active space	Impurity cluster	CASSCF	NEVPT2	tPBE	tPBE0	Literature
*S*_0_ → *T*_1_	(2,2)	OV+Mg_5_	1.67	2.14	2.19	2.06	
		OV+Mg_5_O_8_	1.68	2.30	2.24	2.10	
		OV+Mg_5_O_12_	1.67	2.31	2.25	2.11	
		Extrap	1.68	2.37	2.27	2.12	
	Mg_36_O_36_	OV+Mg_5_	2.23	2.22	2.33	2.31	
		OV+Mg_5_O_8_	2.26	2.28	2.39	2.36	1.93 (MRCI)^68^
		OV+Mg_5_O_1_2	2.27	2.29	2.40	2.37	
		Extrap	2.28	2.30	2.42	2.39	
*S*_0_ → *S*_1_	(2,2)	OV+Mg_5_	3.91	3.76	2.84	3.11	
		OV+Mg_5_O_8_	3.85	3.62	2.93	3.16	
		OV+Mg_5_O_1_2	3.84	3.59	2.93	3.16	
		Extrap	3.82	3.54	2.96	3.15	
	(2,8)	OV+Mg_5_	3.91	3.76	2.84	3.11	3.24 (MRCI)^68^
		OV+Mg_5_O_8_	3.85	3.62	2.93	3.16	2.30 (Exp)^65^
		OV+Mg_5_O_1_2	3.84	3.59	2.93	3.16	1.0, 1.3, 2.4, 3.4 (Exp)^66^
		Extrap	3.82	3.54	2.96	3.15	1.2, 3.6, 5.3 (Exp)^67^

a The extrapolated CAS-DMET, NEVPT2-DMET, tPBE-DMET, and tPBE0-DMET energies from the linear regression are labeled as “Extrap”.

**Figure 7:**
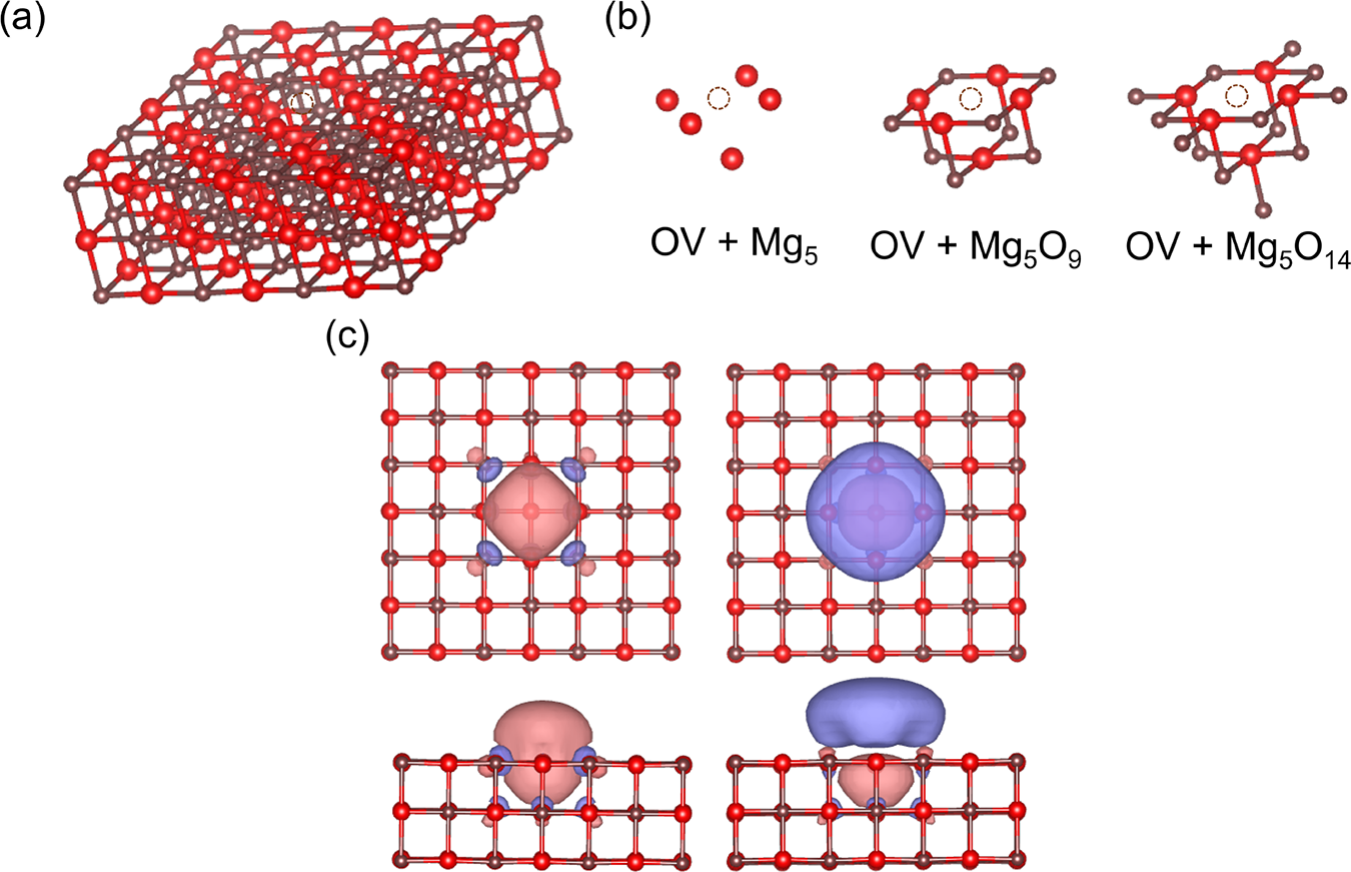
Oxygen monovacancy on a Mg_54_O_54_ layer: (a) F-center on the (100) surface. (b) Four different impurity clusters considered in the DMET calculations. (c) Top and side views of four defect natural orbitals from the converged CAS-DMET calculation considered in the (2, 2) active space. The isosurface of orbitals is 0.02.

In the three-layer case, like in the example above, the nonembedding calculations are prohibitively costly. The active spaces used are (2,2) and (2,8). The computational model used for the OV defect in 3 layers of MgO (Mg_54_O_54_), the impurity clusters used in the embedding calculations, and the natural active orbitals in the minimal (2,2) active space are shown in Figure 7a–c, respectively. As in the two-layer case, the natural orbitals shown here in Figure 7c are obtained from the largest converged CAS-DMET calculations.

**Figure 8:**
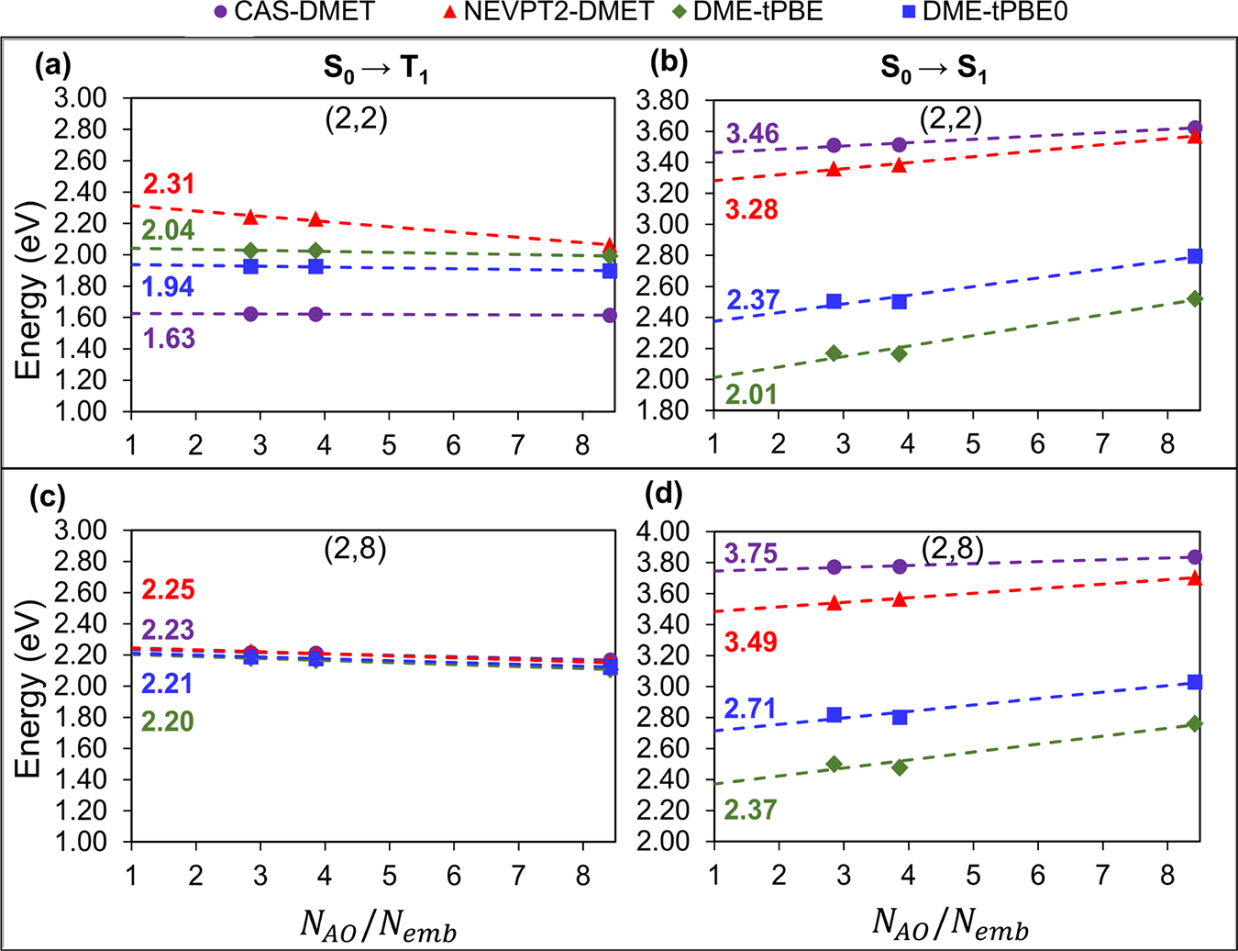
Excitation energies of the OV defect in the Mg_54_O_54_ surface using an ROHF bath and active spaces of (2,2) and (2,8) calculated by CAS-DMET (purple circles), NEVPT2-DMET (red triangles), pDME-tPBE (dark green diamonds), and pDME-tPBE0 (blue squares) for S_0_ → T_1_ (a, c) and S_0_ → S_1_ (b, d) excitations as a function of *N*_AO_/*N*_emb_. All energies are extrapolated to the nonembedding limit; *N*_AO_ represents the number of basis functions, and *N*_emb_ is the number of embedding orbitals. Here *N*_AO_ is 1482.

The vertical excitation energies for the OV defect in Mg_54_O_54_ obtained from the embedding calculations are plotted in Figure 8 and reported in Table 4. Like in the Mg_36_O_36_case, for the (2,2) active space, pDME-tPBE, pDME-tPBE0, and NEVPT2-DMET increase the S_0_ → T_1_ excitation and decrease the S_0_ → S_1_ excitation compared with CAS-DMET. Interestingly, NEVPT2-DMET and pDME-tPBE0 disagree with each other in the S_0_ → S_1_ excitation energy, by 0.76 and 0.64 eV for the (2,2) and (2,8) active spaces, respectively.

**Table 4:** Vertical Excitation Energies (in eV) of the Oxygen Monovacancy on the Mg_54_O_54_ Surface Obtained Using DMET with CASSCF, NEVPT2, MC-PDFT (tPBE), and HMC-PDFT (tPBE0)^a^

Excitation	Active Space	Impurity cluster	CASSCF	NEVPT2	tPBE	tPBE0	Literature
*S*_0_ → *T*_1_	Mg_54_O_54_	OV+Mg_5_	1.61	2.06	1.99	1.90	
		OV+Mg_5_O_8_	1.62	2.23	2.03	1.93	
		OV+Mg_5_O_1_2	1.62	2.24	2.03	1.93	
		Extrap	1.63	2.31	2.04	1.94	
	Mg_54_O_54_	OV+Mg_5_	2.17	2.15	2.11	2.12	
		OV+Mg_5_O_8_	2.21	2.21	2.17	2.18	1.93 (MRCI)^68^
		OV+Mg_5_O_13_	2.21	2.22	2.18	2.19	
		Extrap	2.23	2.25	2.21	2.20	
*S*_0_ → *S*_1_	Mg_54_O_54_	OV+Mg_5_	3.62	3.57	2.52	2.80	
		OV+Mg_5_O_8_	3.51	3.38	2.16	2.50	
		OV+Mg_5_O_13_	3.51	3.36	2.17	2.51	
		Extrap	3.46	3.28	2.01	2.37	
	Mg_54_O_54_	OV+Mg_5_	3.84	3.70	2.76	3.03	3.24 (MRCI)^68^
		OV+Mg_5_O_8_	3.78	3.57	2.48	2.80	2.30 (Exp)^65^
		OV+Mg_5_O_13_	3.77	3.54	2.50	2.82	1.0, 1.3, 2.4, 3.4 (Exp)^66^
		Extrap	3.65	3.30	2.38	2.70	1.2, 3.6, 5.3 (Exp)^67^

a The extrapolated CAS-DMET, NEVPT2-DMET, tPBE-DMET, and tPBE0-DMET energies from the linear regression are labeled as “Extrap”.

## 5 Conclusion

We developed a new electronic stucture method, called pDME-PDFT, based on density matrix embedding theory and multiconfiguration pair-density functional theory, able to treat extended systems with periodic boundary conditions. Initial applications on oxygen vacancies in magnesium oxide showed that produced results are comparable to the more expensive nonembedded MC-PDFT method. We then used pDME-PDFT to study larger models, namely, the Mg_36_O_36_ and Mg_54_O_54_ surfaces, which are impractical to investigate with nonembedded MC-PDFT. Finally, pDME-PDFT gives results comparable with the more expensive and in many cases nonaffordable NEVPT2-DMET method. We envision that pDME-PDFT will be used to investigate the electronic properties of defects in materials, as well as reactions on surfaces involving multireference systems.

## Supporting Information Available

Impurity cluster analysis for the M-center defect in the MgO monolayer; slopes for the linear extrapolations, total energies in hartree for supercells considered for OV in Mg(100) surface for the 2,2 and 2,8 active spaces, total energies for supercells considered for OOV in the Mg(100) surface for 4,5 active space; different (2,8) active spaces explored within this work for OV in the Mg(100) surface; and sample script to run a pDME-PDFT calculation (PDF)
Optimized crystal structure CIF files (ZIP)
