# Supplementary material for: Density Matrix Embedding Using Multiconfiguration Pair-Density Functional Theory
Source: J Chem Theory Comput. 2023 Jun 6;19(12):3498–508. doi: 10.1021/acs.jctc.3c00247 (PMC10308814; doi:10.1021/acs.jctc.3c00247)
Supplement: Supplementary file 1 — ct3c00247_si_001.pdf [file ct3c00247_si_001.pdf]

Supporting Information: Density Matrix Embedding Using Multiconfiguration Pair-Density Functional Theory

Abhishek Mitra,[†] Matthew R. Hermes,[†] and Laura Gagliardi[*,‡,¶]

†*Department of Chemistry, Chicago Center for Theoretical Chemistry, University of Chicago, Chicago, IL 60637, USA.*

‡*Department of Chemistry, Pritzker School of Molecular Engineering, James Franck Institute, Chicago Center for Theoretical Chemistry, University of Chicago, Chicago, IL 60637, USA.*

¶*Argonne National Laboratory 9700 S. Cass Avenue Lemont, IL 60439*

E-mail: lgagliardi@uchicago.edu

Contents

S01 Impurity Cluster Analysis for M-center Defect in MgO Monolayer **S-3**

S02 Slopes for the linear extrapolations **S-5**

S03 Total energies in hartree for supercells considered for OV in Mg(100) surface for 2,2 active space **S-6**

S04 Total energies for supercells considered for OV in Mg(100) surface for 2,8 active space **S-7**

S05 Total energies for supercells considered for OOV in Mg(100) surface for 4,5 active space **S-8**

S06 (2,8) active spaces explored within this work **S-8**

S07 Sample script to run a DME-PDFT calculation **S-9**

S01. Impurity Cluster Analysis for M-center Defect in MgO Monolayer

In this section, we delve deeper into the selection of impurity clusters in the DME-PDFT calculations for the M-center (OOV defect) on the (100) monolayer of MgO. We demonstrate that the OOV+Mg_6 impurity cluster is inadequate in capturing the overall system densities, not due to the number of orbitals but rather the shape of the selected impurity cluster. To address this issue, we increase the number of MLWFs on each atom by maintaining the atoms in the impurity cluster constant, resulting in the same number of MLWFs as in the OOV+Mg_6O_2 impurity cluster. In Figure S1, we compare the $S_0 \rightarrow T_1$ transition between the OOV+Mg_6 and OOV+Mg_6O_2 impurity clusters and a linear extrapolation. The gray circle represents a larger number of MLWFs, while the red circle refers to the default number of MLWFs.

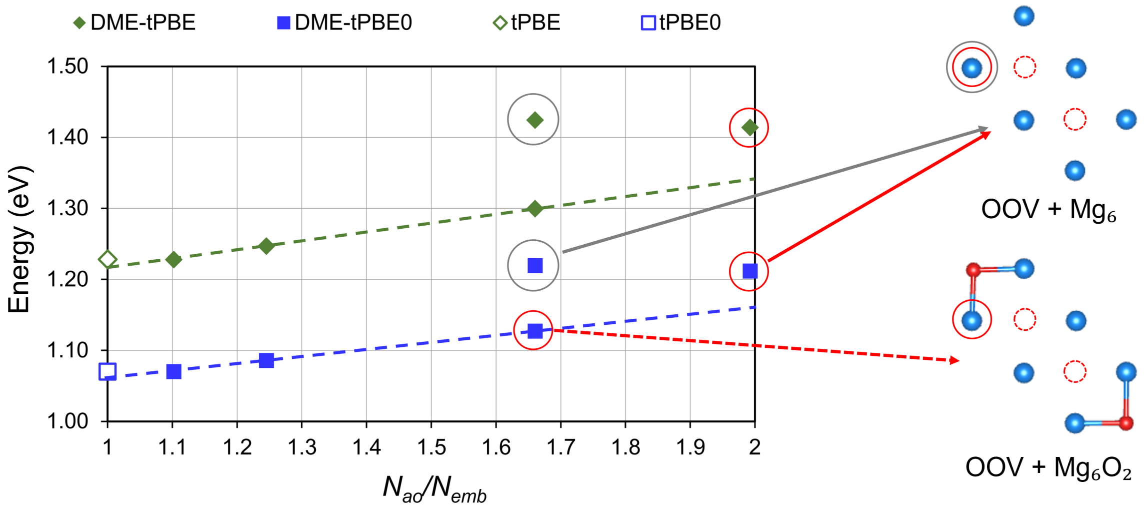

Figure S1: Comparison of $S_0 \rightarrow T_1$ Transition for OOV+Mg_6 and OOV+Mg_6O_2 Impurity Clusters and Linear Extrapolation in MgO Monolayer M-center Defect Calculation. Methods utilized are pDME-tPBE (dark green diamonds) and pDME-tPBE0 (blue squares). The red circles represent the default number of MLWFs, while the gray circle indicates an expanded number of MLWFs. The y-axis has been zoomed in to highlight differences.

To better understand the results, we present the R-squared values obtained from linear extrapolation across all four impurity subspaces (labeled as R-squared-4 points) and the

three largest impurity clusters (labeled as R-squared-3 points). Our results reveal that in the case of the $S_0 \rightarrow T_1$ excitation for the DME-PDFT methods, the inclusion of the smallest impurity cluster decreases the R-squared values from an almost perfect 1.00 to approximately 0.95. Remarkably, we observe significant reductions in the R-squared values for almost all the methods when the OOV+Mg_6 is added to the three largest impurity clusters. All of these results suggest that the smallest impurity cluster should not be included while extrapolating to the non-embedding limit.

Table S1: R^2 values for the linear extrapolations of vertical excitation energies in the oxygen divacancy on the MgO(100) surface obtained using CAS-DMET, NEVPT2-DMET, pDME-tPBE and pDME-tPBE0.

Excitation	Methods	R^2-4 pts	R^2-3 pts
$S_0 \rightarrow T_1$	CAS-DMET	0.47	0.99
	NEVPT2-DMET	0.16	0.88
	DME-tPBE	0.94	1.00
	DME-tPBE0	0.95	1.00
$S_0 \rightarrow S_1$	CAS-DMET	0.99	1.00
	NEVPT2-DMET	0.04	1.00
	DME-tPBE	0.00	0.96
	DME-tPBE0	0.04	0.97

S02. Slopes for the linear extrapolations

Table S2: Slopes for the linear extrapolations (in eV) of the oxygen vacancy on the MgO(100) surface obtained using CAS-DMET, NEVPT2-DMET, pDME-tPBE and pDME-tPBE0.

Excitation	Layers	Active Space	CASSCF	NEVPT2	tPBE	tPBE0
$S_0 \rightarrow T_1$	$Mg_{18}O_{18}$	(2,2)	0.01	0.09	-0.01	0.00
		(2,8)	0.01	0.04	-0.03	-0.02
	$Mg_{36}O_{36}$	(2,2)	0.02	0.07	-0.04	-0.02
		(2,8)	0.02	0.05	-0.03	-0.02
	$Mg_{54}O_{54}$	(2,2)	0.02	0.04	0.07	0.06
		(2,8)	0.01	0.03	0.05	0.04
$S_0 \rightarrow S_1$	$Mg_{18}O_{18}$	(2,2)	-0.01	-0.12	0.03	0.02
		(2,8)	-0.03	-0.06	0.00	-0.01
	$Mg_{36}O_{36}$	(2,2)	0.00	-0.05	-0.02	-0.01
		(2,8)	-0.01	-0.02	-0.02	-0.02
	$Mg_{54}O_{54}$	(2,2)	0.00	-0.03	-0.01	-0.01
		(2,8)	-0.01	-0.01	-0.01	-0.01

Table S3: Slopes for the linear extrapolations (in eV) of the oxygen divacancy on the MgO(100) surface obtained using CAS-DMET, NEVPT2-DMET, pDME-tPBE and pDME-tPBE0.

Excitation	Layers	Active Space	CASSCF	NEVPT2	tPBE	tPBE0
$S_0 \rightarrow T_1$	$Mg_{18}O_{18}$	(4,5)	0.02	0.14	0.13	0.10
$S_0 \rightarrow S_1$	$Mg_{18}O_{18}$	(4,5)	0.04	0.04	0.11	0.09

S03. Total energies in hartree for supercells considered for OV in Mg(100) surface for 2,2 active space

Table S4: Total Energies for the (2,2) active space for the singlet states

State	Layers	Impurity cluster	CASSCF	NEVPT2	tPBE	tPBE0
S_0	$Mg_{18}O_{18}$	OV+Mg_4	-1390.269452	-1390.471724	-1411.899236	-1406.491790
		OV+Mg_4O_4	-1390.269906	-1391.324931	-1411.897388	-1406.490517
		OV+Mg_4O_8	-1390.270106	-1392.436688	-1411.897809	-1406.490883
		Full	-1390.270468	-1394.225515	-1411.897576	-1406.490799
	$Mg_{36}O_{36}$	OV+Mg_5	-2795.546970	-2795.803836	-2840.579608	-2829.321449
		OV+Mg_5O_8	-2795.547167	-2797.491295	-2840.581271	-2829.322745
		OV+Mg_5O_{12}	-2795.547107	-2798.321520	-2840.581652	-2829.323015
	$Mg_{54}O_{54}$	OV+Mg_5	-4200.865578	-4201.095403	-4269.395603	-4252.263097
		OV+Mg_5O_8	-4200.865861	-4202.804838	-4269.396895	-4252.264137
		OV+Mg_5O_{13}	-4200.865908	-4203.854095	-4269.396952	-4252.264191
S_1	$Mg_{18}O_{18}$	OV+Mg_4	-1390.149330	-1390.355352	-1411.806407	-1406.392137
		OV+Mg_4O_4	-1390.150247	-1391.212699	-1411.804907	-1406.391242
		OV+Mg_4O_8	-1390.150670	-1392.326382	-1411.804311	-1406.390901
		Full	-1390.150927	-1394.116920	-1411.803899	-1406.390656
	$Mg_{36}O_{36}$	OV+Mg_5	-2795.412040	-2795.670497	-2840.486527	-2829.217905
		OV+Mg_5O_8	-2795.414675	-2797.365006	-2840.483571	-2829.216347
		OV+Mg_5O_{12}	-2795.415226	-2798.196389	-2840.484193	-2829.216951
	$Mg_{54}O_{54}$	OV+Mg_4O_4	-4200.732371	-4200.964133	-4269.302984	-4252.160331
		OV+Mg_5O_8	-4200.736696	-4202.680450	-4269.3173334	-4252.17217
		OV+Mg_5O_{13}	-4200.736863	-4203.730575	-4269.317213	-4252.172134

Table S5: Total energies in hartree for the (2,2) active space for the triplet state

State	Layers	Impurity cluster	CASSCF	NEVPT2	tPBE	tPBE0
T_1	$Mg_{18}O_{18}$	OV+Mg_4	-1390.221524	-1390.401384	-1411.822598	-1406.422330
		OV+Mg_4O_4	-1390.221517	-1391.247959	-1411.822603	-1406.422331
		OV+Mg_4O_8	-1390.221517	-1392.358635	-1411.822615	-1406.422340
		Full	-1390.221517	-1394.144970	-1411.822590	-1406.422322
	$Mg_{36}O_{36}$	OV+Mg_5	-2795.485566	-2795.725160	-2840.499060	-2829.245686
		OV+Mg_5O_8	-2795.485580	-2797.406934	-2840.498969	-2829.245622
		OV+Mg_5O_{12}	-2795.485581	-2798.236664	-2840.498802	-2829.245507
	$Mg_{54}O_{54}$	OV+Mg_5	-4200.806255	-4201.019622	-4269.322394	-4252.193359
		OV+Mg_5O_8	-4200.806261	-4202.722919	-4269.322409	-4252.193372
		OV+Mg_5O_{13}	-4200.806261	-4203.771728	-4269.322478	-4252.193424

S04. Total energies for supercells considered for OV in Mg(100) surface for 2,8 active space

Table S6: Total Energies in hartree for the (2,8) active space for the singlet states

Excitation	Layers	Impurity cluster	CASSCF	NEVPT2	tPBE	tPBE0
S_0	$Mg_{18}O_{18}$	OV+Mg_4	-1390.296362	-1390.473931	-1411.905389	-1406.503132
		OV+Mg_4O_4	-1390.298224	-1391.323592	-1411.905107	-1406.503386
		OV+Mg_4O_8	-1390.298414	-1392.433991	-1411.905187	-1406.503493
		Full	-1390.298716	-1394.221833	-1411.904093	-1406.502748
	$Mg_{36}O_{36}$	OV+Mg_5	-2795.570239	-2795.804472	-2840.582919	-2829.329749
		OV+Mg_5O_8	-2795.571965	-2797.483251	-2840.585817	-2829.332354
		OV+Mg_5O_{12}	-2795.572152	-2798.312710	-2840.585732	-2829.332337
	$Mg_{54}O_{54}$	OV+Mg_5	-4200.888377	-4201.096917	-4269.397952	-4252.270558
		OV+Mg_5O_8	-4200.890456	-4202.797059	-4269.399523	-4252.272256
		OV+Mg_5O_{10}	-4200.890667	-4203.845255	-4269.399972	-4252.272646
S_1	$Mg_{18}O_{18}$	OV+Mg_4	-1390.168531	-1390.350116	-1411.791260	-1406.385578
		OV+Mg_4O_4	-1390.170918	-1391.201030	-1411.789722	-1406.385021
		OV+Mg_4O_8	-1390.171459	-1392.312701	-1411.788938	-1406.384568
		Full	-1390.171727	-1394.101185	-1411.788734	-1406.384482
	$Mg_{36}O_{36}$	OV+Mg_5	-2795.426650	-2795.666127	-2840.478510	-2829.215545
		OV+Mg_5O_8	-2795.430332	-2797.350327	-2840.478165	-2829.216206
		OV+Mg_5O_{12}	-2795.430938	-2798.180824	-2840.478187	-2829.216375
	$Mg_{54}O_{54}$	OV+Mg_5	-4200.747368	-4200.960788	-4269.296510	-4252.159224
		OV+Mg_5O_8	-4200.751689	-4202.666000	-4269.308493	-4252.169292
		OV+Mg_5O_{13}	-4200.752053	-4203.715057	-4269.308107	-4252.169094

Table S7: Total energies in hartree for the (2,8) active space for the triplet state

Excitation	Layers	Impurity cluster	CASSCF	NEVPT2	tPBE	tPBE0
T_1	$Mg_{18}O_{18}$	OV+Mg_4	-1390.225414	-1390.401149	-1411.819254	-1406.420794
		OV+Mg_4O_4	-1390.225793	-1391.247659	-1411.819004	-1406.420701
		OV+Mg_4O_8	-1390.225856	-1392.357473	-1411.818956	-1406.420681
		Full	-1390.225868	-1394.143431	-1411.818895	-1406.420639
	$Mg_{36}O_{36}$	OV+Mg_5	-2795.488157	-2795.722750	-2840.497129	-2829.244886
		OV+Mg_5O_8	-2795.488798	-2797.399607	-2840.497900	-2829.245625
		OV+Mg_5O_{12}	-2795.488880	-2798.228674	-2840.497423	-2829.245287
	$Mg_{54}O_{54}$	OV+Mg_5	-4200.808742	-4201.017915	-4269.320515	-4252.192572
		OV+Mg_5O_8	-4200.809185	-4202.715894	-4269.319910	-4252.192229
		OV+Mg_5O_{13}	-4200.809276	-4203.763572	-4269.319874	-4252.192224

S05. Total energies for supercells considered for OOV in Mg(100) surface for 4,5 active space

Table S8: Total Energies in hartree for the (4,5) active space for the singlet states

Excitation	Layers	Impurity cluster	CASSCF	NEVPT2	tPBE	tPBE0
S_0	$Mg_{18}O_{18}$	OOV+Mg_6	-1374.632877	-1374.936190	-1395.747900	-1390.469144
		OOV+Mg_6O_2	-1374.634140	-1375.456204	-1395.746892	-1390.468704
		OOV+Mg_6O_6	-1374.635109	-1376.931410	-1395.727138	-1390.454131
		OOV+Mg_6O_{10}	-1374.635334	-1377.683659	-1395.729493	-1390.455954
		Full	-1374.635636	-1378.416978	-1395.729166	-1390.455783
S_1	$Mg_{18}O_{18}$	OOV+Mg_6	-1374.585159	-1374.884328	-1395.690491	-1390.414158
		OOV+Mg_6O_2	-1374.586734	-1375.402901	-1395.686870	-1390.411836
		OOV+Mg_6O_6	-1374.588355	-1376.879000	-1395.669140	-1390.398944
		OOV+Mg_6O_{10}	-1374.588758	-1377.631189	-1395.671615	-1390.400901
		Full	-1374.589139	-1378.364610	-1395.671380	-1390.400820

Table S9: Total energies in hartree for the (4,5) active space for the triplet state

Excitation	Layers	Impurity cluster	CASSCF	NEVPT2	tPBE	tPBE0
T_1	$Mg_{18}O_{18}$	OOV+Mg_6	-1374.610634	-1374.902884	-1395.695936	-1390.424611
		OOV+Mg_6O_2	-1374.611709	-1375.416939	-1395.699129	-1390.427274
		OOV+Mg_6O_6	-1374.612957	-1376.895024	-1395.681317	-1390.414227
		OOV+Mg_6O_{10}	-1374.613339	-1377.646945	-1395.684373	-1390.416614
		Full	-1374.613756	-1378.379654	-1395.684032	-1390.416463

S06. (2,8) active spaces explored within this work

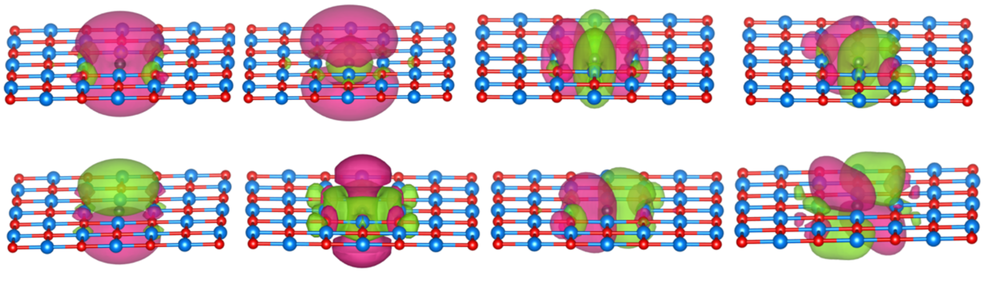

Figure S2: 2,8 active orbitals for single layer $Mg_{18}O_{18}$ unit cell

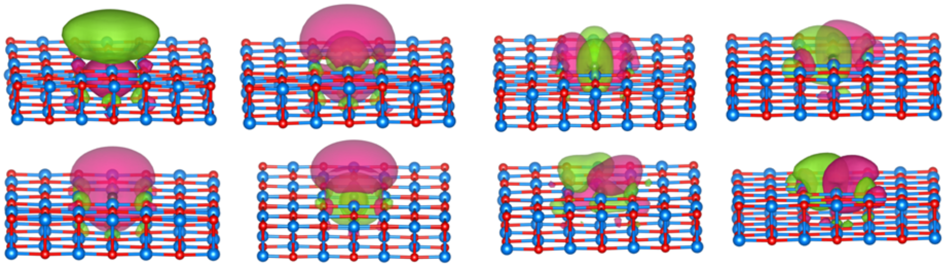

Figure S3: 2,8 active orbitals for double layer $Mg_{36}O_{36}$ unit cell

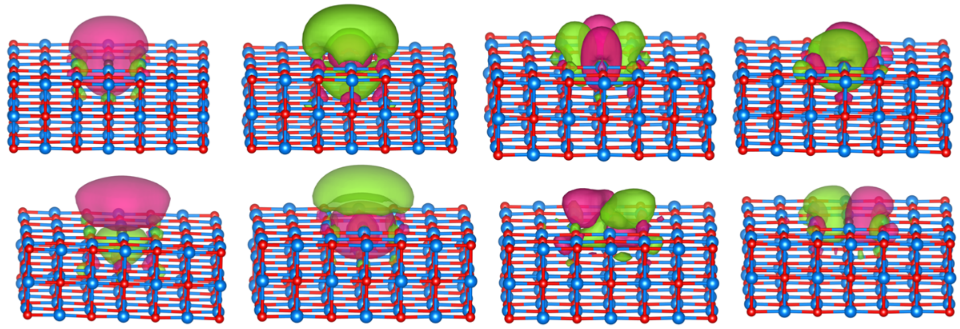

Figure S4: 2,8 active orbitals for triple layer $Mg_{54}O_{54}$ unit cell

S07. Sample script to run a DME-PDFT calculation

This script carries out a pDME-PDFT calculation on the O9Mg5 impurity cluster located on the $Mg_{54}O_{54}$ surface. It begins by defining the unit cell with the help of the mcu library and importing various required libraries and modules, including the custom-built pdmet module developed by the authors. Next, the script builds the GDF, performs the RHF calculation, generates the MLWFs using PyWannier90, and finally executes the pDME-PDFT calculation through the pdmet module.

```
import os, sys, re, mcu
import numpy as np
from pyscf.pbc import gto, scf, cc, df
import pywannier90
from pdmet import dmet
```

```
from pdmet.tools import tchkfile

'''==================================='''
''' Definte unit cell '''
'''==================================='''
a, atoms = mcu.CELL().cif2pyscf('/path/OVC_MgO.cif')
atoms.append(['X:O', [6.16056842, 6.16056842, 11.113246159135699]])
for i, atm in enumerate(atoms):
    if atm[0] == 'Mg' and np.linalg.norm(atm[1] - atoms[-1][1]) < 2.5:
        atoms[i][0] = "Mg*"

cell = gto.Cell()
cell.atom = atoms
cell.basis = {'Mg':'gth-dzvp', 'O':'gth-dzvp',
'Mg*':'gth-tzvp', 'X-O':'gth-tzvp'}
cell.pseudo = 'gth-pbe'
cell.a = a
cell.spin = 2
cell.verbose = 5
cell.build()

'''==================================='''
''' Build GDF '''
'''==================================='''
kmesh = [1, 1, 1]
kpts = cell.make_kpts(kmesh)
if not os.path.exists('gdf.h5'):
```

```
    gdf = df.GDF(cell, kpts)
    gdf._cderi_to_save = 'gdf.h5'
    gdf.build()

'''===================================='''
''' Run RHF '''
'''===================================='''
khf = scf.KROHF(cell, kpts).density_fit()
khf.with_df._cderi = 'gdf.h5'
khf.chkfile = 'chkfile'
khf.init_guess = 'chkfile'
khf.exxdiv = None
khf.run()
tchkfile.save_kmf(khf, 'chk_HF')

'''===================================='''
''' Contruct MLWFs '''
'''===================================='''
kmf = tchkfile.load_kmf(cell, khf, kmesh, 'chk_HF')
num_wann = cell.nao
keywords = \
'''
dis_froz_max = 0.0
begin projections
random
O:s,p,d
Mg:s,p,d
```

```
end projections
num_iter = 1000
'''

w90 = pywannier90.W90(kmf, cell, kmesh, num_wann, other_keywords=keywords)
w90.kernel()
w90.plot_wf(outfile='./WFs/MLWF')
tchkfile.save_w90(w90, 'chk_w90')
ham = w90.get_hamiltonian_kpts()
e, v = np.linalg.eigh(ham)


'''=========================================='''
''' Run DMET '''
'''=========================================='''
pdmet = dmet.pDMET(cell, kmf, w90, solver = 'SA-CASPDFT')
pdmet.impCluster = [108, 72,84,88,98,   23,24,39,40, 27,31,33,36, 86]
pdmet._impOrbs_threshold = 1.6
pdmet.kmf_chkfile = '../rohf/chk_HF'
pdmet.w90_chkfile = '../rohf/chk_w90'
pdmet.twoS = 0
pdmet.cas = (2,2)
pdmet.molist = [190,191]
pdmet.e_shift = 0.5
pdmet.state_average_ = [0.5, 0.5]
pdmet.nevpt2_roots = [0, 1, 2, 3]
pdmet.nevpt2_nroots = 4
pdmet.initialize()
```

```python
pdmet.one_shot()
print("Occupancy:", pdmet.qcsolver.mc.mo_occ)
pdmet.plot('nat', path='./nat')
```
